# Visual and olfactory associative learning in the malaria vector *Anopheles gambiae *sensu stricto

**DOI:** 10.1186/1475-2875-11-27

**Published:** 2012-01-27

**Authors:** Nora Chilaka, Elisabeth Perkins, Frédéric Tripet

**Affiliations:** 1 Centre for Applied Entomology and Parasitology, School of Life Sciences, Keele University, Keele, Staffordshire ST5 5BG, Newcastle, UK

## Abstract

**Background:**

Memory and learning are critical aspects of the ecology of insect vectors of human pathogens because of their potential effects on contacts between vectors and their hosts. Despite this epidemiological importance, there have been only a limited number of studies investigating associative learning in insect vector species and none on Anopheline mosquitoes.

**Methods:**

A simple behavioural assays was developed to study visual and olfactory associative learning in *Anopheles gambiae*, the main vector of malaria in Africa. Two contrasted membrane qualities or levels of blood palatability were used as reinforcing stimuli for bi-directional conditioning during blood feeding.

**Results:**

Under such experimental conditions *An. gambiae *females learned very rapidly to associate visual (chequered and white patterns) and olfactory cues (presence and absence of cheese or Citronella smell) with the reinforcing stimuli (bloodmeal quality) and remembered the association for up to three days. Associative learning significantly increased with the strength of the conditioning stimuli used. Importantly, learning sometimes occurred faster when a positive reinforcing stimulus (palatable blood) was associated with an innately preferred cue (such as a darker visual pattern). However, the use of too attractive a cue (e.g. Shropshire cheese smell) was counter-productive and decreased learning success.

**Conclusions:**

The results address an important knowledge gap in mosquito ecology and emphasize the role of associative memory for *An. gambiae*'s host finding and blood-feeding behaviour with important potential implications for vector control.

## Background

As in many animals, memory and learning are fundamental aspects of the ecology of insect species. Visual place learning is critical for foraging and homing in most insects [1-4] and is most conspicuously demonstrated in the social insects [5,6]. Olfactory learning is equally important as it enables insects to optimize foraging and other activities in their local environment and has also been reported in several insect species [7-9]. Understanding learning and memory bears particular importance for mosquitoes, sandflies, kissing bugs and other insects that transmit human diseases because their behaviour, particularly in relation to host finding and bloodmeal acquisition, may have critical implication for their vectorial capacity and thus the epidemiology of the diseases they transmit (reviewed in [10,11]).

Despite its importance, there is a relative paucity of experimental studies focusing on memory in medically-important insect species and much of the evidence stems from descriptive studies in which non-associative learning or habituation cannot be distinguished from associative learning [11]. Habituation consists in the progressive decrease in the intensity of a pre-existing behavioural response to a stimulus that is neither particularly rewarding nor harmful. A classic example of habituation in insects is found in the Californian desert seed-harvesting ants *Pheidole tucsonica *and *P. gilvescens*, which were shown to decrease their level of aggression towards individuals from neighbouring colonies following repeated exposures to their odours [12]. Habituation is thus a simple process enabling an insect's familiarization with its environment and an adjustment of its behavioural responses to it.

In contrast, associative learning or conditioning occurs when an unpaired stimulus or cue (e.g. odour or visual pattern) becomes paired with a separate reinforcing or conditioning stimulus consisting in a benefit/reward or a cost/punishment leading to a behavioural change or the development of a new behavioural process altogether [13]. For example, in bi-directional conditioning experiments with cockroaches, individuals presented with a peppermint smell associated with the reward of sugar water and vanilla odour associated with unpalatable quinine water, learned these associations and subsequently approached peppermint and avoided vanilla when exposed to these odours [7]. Using the same positive (sugar water) and negative (quinine water) reinforcing stimuli, carpenter ants were trained to remember odours associated with them for up to three days and approached or avoided them accordingly [14].

Evidence for learning and memory in mosquitoes stems from observations that in some species, males tend to exhibit swarm site fidelity and that in mark-release-recapture studies, females were sometimes recaptured in the location in which they were first captured (e.g. [15-18]). However, as critically reviewed by Alonso and Schuck [11], in most of these studies it is impossible to determine whether these few individuals exhibit site fidelity because of habituation, simply by chance, or because of characteristics of the environment itself. Indeed, because mosquitoes are expected to be found in parts of their habitat suitable for mating, oviposition, feeding, resting, etc., they are also more likely to be recaptured in those very same areas. Hence the non-random distribution of mosquitoes over habitats and their non-random exploitation of resources within habitats inevitably leads to some degree of apparent site-fidelity in recapture studies. In other studies, the design of the experiments did not allow for differentiating learning from possible innate preferences linked to different mosquito genetic backgrounds (e.g. [19,20]).

Oviposition-site fidelity suggestive of olfactory memory has been reported in two studies in which chemicals with distinct smells were added to the larval rearing water of *Culex quinquefasciatus *[20] and *Aedes aegypti *[21] and the subsequent oviposition behaviour of adult females recorded. In both studies, females showed some degree of preference for laying eggs in the water they were reared in thereby supporting some process of pre-acquired knowledge of their former larval habitat characteristics. However, non-associative learning through familiarization or habituation is likely to be the process involved rather than associative learning [11].

Since Alonso and Schuck-Palm's [11] critical assessment of the evidence for memory in mosquitoes, associative learning has been specifically and directly tested in at least two experimental studies [22,23]. Using glass pipettes mounted with a small odour-coated filter paper to present a reward in the form of sugar water or blood as reinforcing stimuli to males and female *Cx quinquefasciatus*, Tomberlin et al. [22] were able to condition them into approaching the odour source and probe it even in the absence of the reward [22]. Finally, larvae of *Culex restuans *were conditioned to associate predator odour (salamander odour) with the alarm cues of crushed conspecifics and subsequently displayed predator avoidance behaviour whenever exposed to the salamander odour [23].

Here, a series of experiments aimed at describing the occurrence, extent and processes of associative memory and learning in *Anopheles gambiae*, the main vector of malaria in Africa, were performed. A simple assay was developed for bi-directional conditioning and testing of memory in groups of female individuals. Female mosquitoes were trained to avoid or be attracted to visual cues by associating these cues with a bloodmeal of low or high quality. Under such experimental conditions, *An. gambiae *learned very rapidly and remembered the association for up to three days. Importantly, associative learning and long-term memory could also be mediated via odour cues, but learning was affected by the type of odour used - i.e. if the odour was inherently attractive or not to female mosquitoes. The importance of these findings for *An. gambiae *vector capacity and their potential implications for vector control are discussed.

## Methods

### Standardized mosquito rearing

A Mopti strain of *Anopheles gambiae *sensu stricto colonized in 2007 from field collections made in the region of N'Gabacoro Droit, in Mali, West Africa and maintained in F. Tripet's laboratory, Keele University was used for all experiments. The experiments themselves took place between 2007 and 2009 and thus involved many mosquito generations and many independent rearing episodes in order to obtain several replicates for each experiment. Mosquitoes were kept at 25°C ± 1°C and 70-80% relative humidity. In order to achieve homogeneity in phenotypic quality, all females used in behavioural studies were reared under standard conditions. Adult females were fed on horse blood using an artificial feeder (Hemotek membrane feeding system, Discovery workshops, UK). Eggs were laid two days post blood-feed and hatched within two days. A day later, newly emerged first instars were distributed into plastic trays (34 × 24 cm) at a density of 200 larvae per tray in 1 l of water. They were fed daily 80 mg of ground fish food (Tetra werk, Mulle, Germany) in order to yield adults of good size and phenotypic quality [24]. Pupae were placed in a standard 5 l rearing cages for emergence and newly emerged male and female mosquitoes were kept together and with access to 5% glucose solution at all time. Standard rearing cages are made of 5 l white polypropylene buckets (~20.5 cm height × 20 cm diameter) with a sleeved side opening for introducing and removing mosquitoes and accessories, and the top covered with mosquito netting.

### Bi-directional conditioning and short-term learning assay

**Figure 1:**
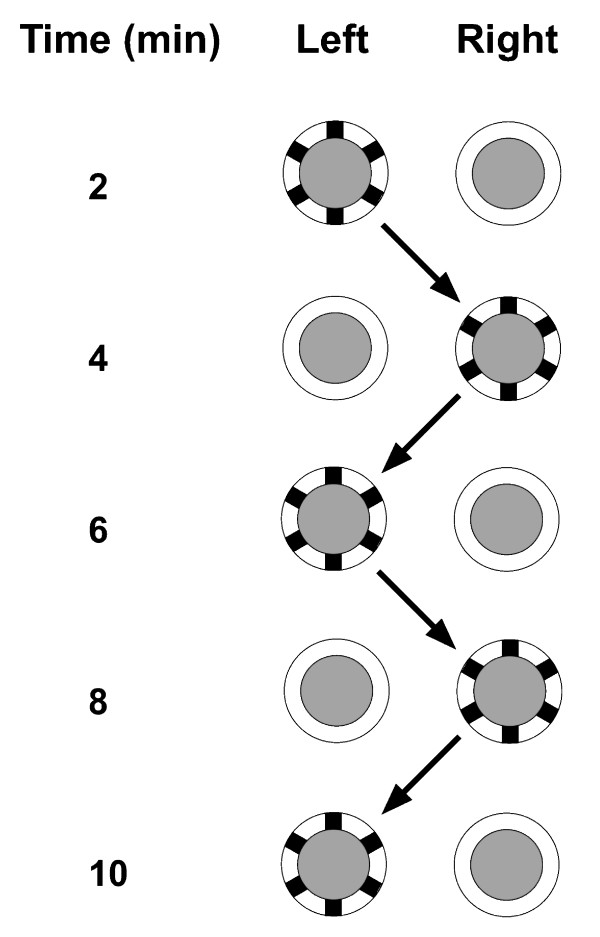
**Experimental set-up for visual conditioning training and testing experiments**. Two blood feeders with either a white or chequered visual pattern around them (underside view depicted here) were placed on the top of a 5 l experimental training/testing cage. Their position was alternated every 2 or 5 min (see text for details) for five times and the number of females on each feeder recorded at each time interval.

In all experiments, 40 two-to-six-day old females which had not yet been blood fed were collected from rearing cages and moved into a training/testing cage. The behavioural training/testing cages consisted in 5 l rearing cages similar to the usual rearing cages, but with a viewing window cut in its side covered by mesh. For all experiments, two Hemotek membrane-feeding system feeders were placed on top of a training/testing cage 5 cm apart. Learning was induced by associating a stimuli (visual, olfactory or other) with the action of landing on the feeder probing and feeding through the use of bi-directional conditioning through two contrasted reinforcing stimuli (reward/punishment) in the form of good and bad membrane qualities or palatable and unpalatable blood (see details below). The unconditioned stimuli and their associated feeder were placed on top of the testing cage and their position switched at a set time interval (2 min for short-term memory and 5 min for long-term memory experiments) for five intervals (Figure 1). Because *An. gambiae *females take ~10-20 min to feed to repletion, switching the position of the feeders interrupted their blood meal and females were thus forced to reposition themselves on the feeders several times in order to obtain a full bloodmeal. Whether the unconditioned stimuli became associated with the reinforcing reward/punishment through learning was recorded by counting the number of mosquitoes that landed on each feeder and initiated feeding using two click-counters.

### Method validation experiments

When performing conditioning/learning assays, in some instances the position of the feeders was switched at the 5th time interval, this time inverting the stimuli/feeder association. This enabled to regularly test whether mosquitoes effectively followed the conditioned stimuli through associative memory or if they used another source of information to directly track their preferred feeder. In addition, these 'method validation' experiments, demonstrated that females did not quickly move between feeders (from bad to good) thereby biasing our recording of their distribution at each time intervals.

### Visual cues

For studies on visual associative memory, two distinct visual stimuli or patterns were made using thick black and white paper. The patterns were shaped as circles that could fit around the two membrane feeders so that they would be clearly visible by mosquitoes resting on the cage walls or flying towards the feeders. Bi-directional visual conditioning was achieved by associating the contrasting colour patterns around the feeders with the action of landing and feeding via manipulation of the reward of the bloodmeal.

At first, contrasting membrane qualities were used: sausage skin, which mosquitoes much prefer, was covered by a layer of Parafilm sealing film (Brand, Wertheim, Germany) and used on one feeder, and a double layer of Parafilm sealing film on the other. Two independent preliminary experiments on membrane preference showed that female *An. gambiae *simultaneously offered blood feeders covered with sausage skin, sausage skin covered with Parafilm, and Parafilm alone, significantly preferred sausage skin over the other two types of membranes and that sausage skin covered by Parafilm was significantly favored over Parafilm alone (Chi-square: *P *< 0.001 in both cases). Sausage skins were defrosted and soaked in water to expand them before use. The membranes was stretched over the blood holder and secured with a rubber collar. Any excess membrane from around the blood holder was then cut away. Both types of membranes were covered with an extra layer of Parafilm to make them appear visually identical. Using a micropipette, 1 ml of horse blood was distributed into each blood feeder. The Hemotek feeding system were set at 37°C ± 1°C for all experiments and the temperature of each feeder double-checked to ensure that they did not differ in temperature.

In subsequent experiments, a much stronger differential reinforcing stimuli was created by drastically manipulating the quality of the bloodmeal contained in one feeder using salt. The blood in one feeder was made unpalatable (40% NaCl) whilst the blood in the other feeder was kept untouched (referred to as unsalted blood or good blood).

### Olfactory cues

For studies on olfactory associative memory, a 1 × 1 cm cube of Blue Shropshire cheese was placed in the bottom of a 2.5 × 5 cm black plastic container (film box). Cheese smell is known to attract mosquitoes because of similarities in odour compounds generated by the bacterial flora found on some cheeses and that living on human feet [25]. Studying the interaction between odour attractants and learning is potentially relevant to the development of mosquito trapping devises (see discussion). Here, a film box with cheese was laid on its side next to the first membrane feeder and a similar but empty film box placed next to the second feeder so that they would look identical to mosquitoes resting on the cage walls or flying towards the feeders.

Another set of experiments was made using Citronella essence, which is thought to act as a repellent against mosquitoes [26]. Studying the interaction between odour repellents and learning is again potentially relevant for the efficacy of such compounds and their application to vector control (see discussion). A few drops of Citronella essence were dispensed on a small piece of cotton wool, which was again place within a film box (as above) laid on its side next to the first membrane feeder. A similar looking but empty film box was placed next to the second feeder.

The Hemotek feeding system was set at 37°C ± 1°C for all experiments and the temperature of each feeder double-checked to ensure that it did not differ. Bi-directional olfactory conditioning was achieved by associating the location of the source of the smell with unpalatable (salted) or good (unsalted) blood.

### Bi-directional conditioning and long-term learning assay

Long-term learning/memory was studied using the exact same experimental designs as short-term learning except for the fact that previously trained mosquitoes were re-assayed 24, 72 and 96 h after their first training/learning session. The initial distribution of mosquitoes on both feeders at the beginning of the training/learning session was then compared statistically to that at the onset of the re-testing session.

### Statistical analyses

All statistical analyses were performed using the software JMP8.02 (SAS Institute, Inc). Pearson Chi-squares (likelihood ratios) were used to test the effect of the reinforcing stimulus position/side (e.g. position of salted blood versus non-salted blood feeders) on the distribution of probing and feeding females at a given time. Logistic regressions were conducted to study the combined effects of time (ordinal variable), the reinforcing stimulus position/side (e.g. position of salted blood versus non-salted blood feeders), the association between the reinforcing stimulus position and the visual or olfactory cue (e.g. salted blood with chequered colour pattern and non-salted blood with white pattern or reverse), and the interaction between these two factors on the number of females on the feeders (dependent variable). Logistic regressions were also conducted separately at given time intervals in order to compare the relative significance of each variable at each interval. The direct effect of replicate (variation in total number of mosquitoes feeding in a given experiment) and its interactions were removed from models except when this significantly affected the resulting model. Similarly all non-significant interactions between variables were manually removed from models using a stepwise procedure.

Long-term memory was simply tested by comparing the number of mosquitoes recorded as probing or feeding on a feeder (dependent variable) at the beginning of the first training/learning session (first time interval) versus the beginning of the next session 24, 72 or 96 h later using logistic regression. The effects of the association between cues and the reinforcing stimuli were also included in these models.

## Results

### Associative visual memory

#### Short-term memory

*Anopheles gambiae *females quickly learned to recognize the chequered and white patterns circling the feeders and to associate that cue with the bi-directional reinforcing stimuli, high and low membrane quality (respectively sausage skin and Parafilm). The combined results of two replicates (chequered pattern with sausage skin and reverse) show that there was no significant difference in the numbers of mosquitoes feeding on each feeder 2 and 4 min after initiating the session, but from 6-10 min, there is a clear and significant preference for the feeder with the sausage skin membrane over that with a Parafilm membrane (Table 1).

**Table 1:** Bi-directional conditioning experiment for short-term visual memory in *Anopheles gambiae *females using contrasted membrane qualities

Association	Chequered/Sausage skin		White/Sausage skin		*χ*^2^	*p*-value
**Membrane**	**Sausage skin**^**†**^	**Parafilm**^**†**^	**Sausage skin**^**†**^	**Parafilm**^**†**^	**-**	**-**

**Time (min)**	***n***	***n***	***n***	***n***		

2	17	15	10	9	0.17	0.674

4	18	10	11	7	3.17	0.075

6	17	8	13	4	7.70	**0.006**

8	15	4	12	4	10.89	**0.001**

10	18	1	14	3	24.79	**< 0.001**

† Both membrane types were covered by an extra layer of Parafilm to ensure similar appearances

The change in number of females on two blood feeders with contrasting membrane qualities (sausage skin or Parafilm) used as reinforcing stimuli associated with chequered and white visual patterns for bi-directional conditioning was recorded. The position of each feeder was alternated every 2 min (see methods for details). Significant results are in bold

#### Effect of reinforcing stimulus strength

**Figure 2:**
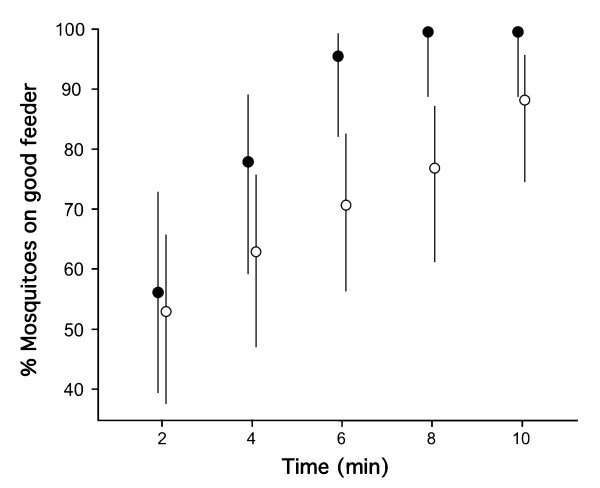
**Effect of the strength of the reinforcing stimulus on learning rate in *Anopheles gambiae *females**. The mean proportion (± 95%CIs) of females probing and feeding on the preferred feeder associated with the positive reinforcement is shown for two sets of experiments. In the first experiment (white circles) the positive and negative reinforcing stimuli were the preferred sausage-skin membrane or the disliked Parafilm membrane; in the second (black circles), unpalatable salted blood and non-salted blood was used (see text and Table 1, 2 for details).

The same experiment conducted with the stronger bi-directional reinforcing stimuli of non-salted and salted blood led to similar results but females showed a significant preference for the feeder with non-salted blood already 4 min after the start of the session (Table 2). Comparing the relatively mild bi-directional reinforcing stimuli of the manipulation of membrane quality versus the drastic manipulation of blood palatability using logistic regression, revealed that the strength of the reinforcing stimuli significantly affected learning rate as evidenced by the strong significant interaction between reinforcing stimulus strength and the variable 'time' on the proportion of females feeding on each feeder (Logistic regression: n = 362, time: ***χ*^2^**= 54.7, *df*= 4, *P *< 0.001; reinforcing stimuli strength: ***χ*^2^**= 0.11, *df*= 1, *p *< 0.745, interaction: ***χ*^2^**= 13.94, *df*= 4, *P*= 0.008) (Figure 2).

**Table 2:** Bi-directional conditioning experiment for short-term visual memory in *Anopheles gambiae *females using contrasted blood qualities

Association	Chequered/Normal blood		White/Normal blood		*χ*^2^	*p*-value
**Blood**	**Normal blood**^†^	**Salted blood**^**†**^	**Normal blood**^**†**^	**Salted blood**^**†**^	**-**	**-**

**Time (min)**	***n***	***n***	***n***	***n***		

2	9	7	8	6	0.5	0.465

4	11	4	10	2	8.8	**0.003**

6	16	1	10	0	28.9	**< 0.001**

8	19	0	15	0	47.1	**< 0.001**

10	18	0	16	0	47.1	**< 0.001**

†The change in number of females on two blood feeders with contrasting bloodmeal quality (salted and normal blood) used as reinforcing stimuli associated with chequered and white visual patterns for bi-directional conditioning. The position of each feeder was alternated every 2 min (see methods for details). Significant results are in bold

#### Effect of innate unpaired stimuli preference

**Figure 3:**
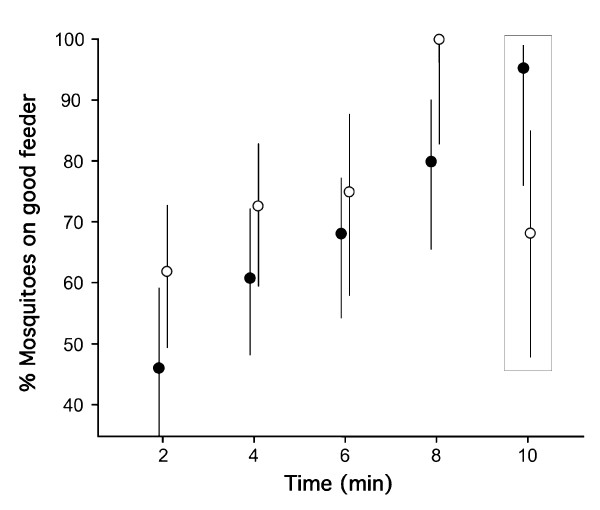
**Effect of innate preference in unpaired visual stimulus on learning rate in *Anopheles gambiae *females**. The mean proportion (± 95%CIs) of females probing and feeding on the preferred feeder associated with the positive reinforcement is shown for two sets of replicates of the same experiment. The positive reinforcing stimulus of non-salted blood was associated with the innately preferred chequered visual pattern in the first set (white circles) and with the less-favoured white pattern in the second (black circles).

Adult female mosquitoes may innately prefer one unpaired stimulus or cue (here colour pattern) over another and, if that pattern is associated with the positive reinforcing stimulus (good feeder) during conditioning, this could affect learning rate. The potential effect of innate cue preference was explored by adding this as a variable 'association' in a logistic regression that included additional replication of the previous experiments (three replicates with association in both directions). Similar to what was found previously, on average females learned to follow the visual stimulus associated with non-salted blood from as early as the second interval (4 min) of the training/testing session to its end (10 min)(Chi-square: *P *< 0.001 in all cases). The direction of the association between colour patterns and reinforcing stimuli had a significant impact on learning. Overall, females preferred the association between chequered colour and non-salted blood than the reverse feeder/colour pattern association throughout the training session as evidenced by a significant 'association' variable in the model (Logistic regression: n = 386, time: ***χ*^2^**= 24.8, *df*= 3, *P *< 0.001; association: ***χ*^2^**= 8.42, *df*= 1, *p*= 0.004, interaction ns). Note that the data from time = 10 min was omitted in this analysis as a few late participating mosquitoes led to an artifactual low value for feeder preference (Figure 3).

### Long-term memory

**Figure 4:**
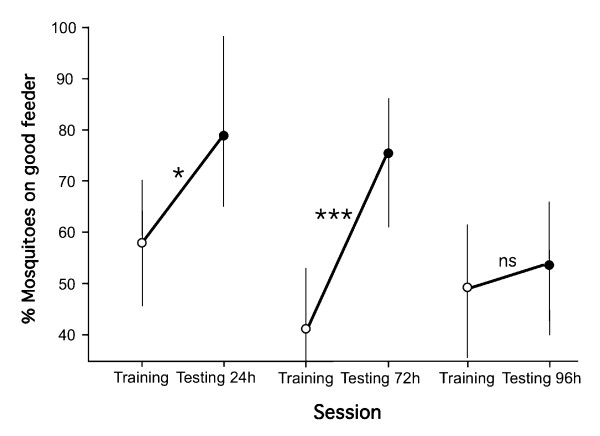
**Long-term memory retention in *Anopheles gambiae *females previously conditioned to associate visual cues with non-salted or salted blood**. The mean proportion (± 95%CIs) of females probing and feeding on the preferred feeder associated with the positive reinforcement (non-salted blood) is shown at the beginning of the training experiment (white circles) when the stimulus is still unpaired, and at the beginning of the re-testing session. Independent experiments tested memory retention after 24 h, 72 h, and 96 h (see test for details). Significance levels are *P *< 0.05 *, *P *< 0.005 **, *P *< 0.001 ***, *P *> 0.05 ns.

Whether trained females remembered the association between a visual pattern and the unsalted blood for long periods of time after conditioning was tested by comparing their initial feeder preference at the beginning of the first training/testing session (untrained females) with their initial feeder preference in a second session (trained females) conducted 24, 72 and 96 h later (three independent experiments replicated three times in both directions for each re-testing intervals). Females significantly preferred the feeder with non-salted blood 24 h after the training session (Chi-square likelihood ratio: *n*= 105, ***χ*^2^**= 4.49, df = 1, *P*= 0.034); their preference was very strong after 72 h (*n*= 119, ***χ*^2^**= 13.25, *p *< 0.001), but disappeared after 96 h (*n*= 111, ***χ*^2^**= 0.23, *P*= 0.636) (Figure 4). Note that the association between colour pattern and salted versus non-salted feeder was not significant at any of those time intervals (Logistic regression: *P *> 0.224 in all cases).

### Method validation experiments

Six replicates of method validation experiments (each involving both directions of the association between cue and reinforcing stimuli) in which the association of cue and conditioning stimuli was inverted in the 5th time interval were run in the course the study to verify that female mosquitoes effectively followed the cue associated with the preferred feeder because of learning. In all such experiments females were fooled into probing/feeding onto the disliked feeder thereby proving that the conditioning/training sessions resulted in effective learning (Combined Chi-square: *P *< 0.001) (Table 3).

**Table 3:** Example of method validation experiment using contrasted blood qualities

Association	Chequered/Normal blood		White/Normal blood		*χ*^2^	*p*-value
**Blood**	**Normal blood**^**†**^	**Salted blood**^**†**^	**Normal blood**^**†**^	**Salted blood**^**†**^	**-**	**-**

**Time (min)**	***n***	***n***	***n***	***n***		

2	14	16	9	7	0.0	1.000

4	19	7	9	4	7.2	**0.006**

6	15	4	16	4	9.9	**0.002**

8	17	0	17	3	30.5	**< 0.001**

10*	**0**	**16**	**1**	**20**	42.1	**< 0.001**

*Here the association between the visual stimulus and blood quality was inverted

†The change in number of females on two blood feeders with contrasting bloodmeal quality (salted and normal blood) associated, in this instance, with chequered and white visual patterns for bi-directional conditioning was recorded. The position of each feeder was alternated every 2 min; but at time 10 min (in bold) the association between visual cue and feeder quality was inverted in order to verify that females effectively followed the cue and did not simply distinguish feeders. Females were thus fooled into probing/feeding on salted blood. Significant results are in bold

### Associative olfactory memory

#### Short-term olfactory memory

*Anopheles. gambiae *females also learned to recognize smells and to associate their intensity with the bi-directional reinforcing stimuli. In a first experiment, the smell of Blue Shropshire cheese was associated with the bi-directional reinforcing stimuli of salted or not salted blood. The combined results of two replicates (strong cheese smell with salted blood and reverse) show that there was no significant difference in the numbers of mosquitoes feeding on each feeder 2, 4 and 6 min after initiating the session, but at 8 (one-way test) and 10 min, there was a significant preference for the feeder with non-salted blood over the salted one (Table 4). The direction of the association between smell and salted/non salted blood significantly affected the distribution of females on the feeders, with the cheese/non-salted blood attracting more females, and this was true for all time intervals (4, 6, 8 and 10 min) except the first one (Logistic regression: *P *< 0.001 in all cases).

**Table 4:** Bi-directional conditioning experiment for short-term olfactory memory to cheese smell in *Anopheles gambiae *females

Association	Cheese/Normal blood		No cheese/Normal blood		*χ*^2^	*p*-value
**Blood**	**Normal blood**^**†**^	**Salted blood**^**†**^	**Normal blood**^**†**^	**Salted blood**^**†**^	**-**	**-**

**Time (min)**	***n***	***n***	***n***	***n***		

2	10	4.5	10.5	9.5	2.46	0.115

4	6.5	1.5	6	9	0.35	0.555

6	7	1	5.5	7	1.99	0.158

8	6	0.5	7	6.5	3.66	0.056*

10	6	0	6.5	4.5	7.84	**0.005**

†The change in number of females on two blood feeders with contrasting bloodmeal quality (salted and normal blood) used as reinforcing stimuli associated with and without Shropshire cheese smell for bi-directional conditioning was recorded. The position of each feeder was alternated every 2 min (see methods for details). Significant results are in bold (two-way test) and labelled with an asterisk (one-way test)

### Effect of different unpaired stimuli

In a second experiment, the smell of Citronella was associated with salted/non-salted blood. The combined results of two replicates (Citronella smell with normal blood and reverse) show that female mosquitoes learned to associate the Citronella smell and blood quality already 4 min after initiating the session and stayed choosey until the end of the session (Table 5). The direction of the association between Citronella smell and salted/non salted blood did not significantly affect the distribution of females on the feeders but there was a significant effect of time, consistent with the results of Table 5 (Logistic regression: *n*= 327, time: ***χ*^2^**= 18.2, *df*= 4, *p*= 0.001, association: ***χ*^2^**= 0.32, *df*= 1, *p*= 0.573, interaction ns).

**Table 5:** Bi-directional conditioning experiment for short-term olfactory memory to Citronella smell in *Anopheles gambiae *females

Association	Citronella/Normal blood		No Citronella/Normal blood		*χ*^2^	*P*-value
**Blood**	**Normal blood**^**†**^	**Salted blood**^**†**^	**Normal blood**^**†**^	**Salted blood**^**†**^	**-**	**-**

**Time (min)**	***n***	***n***	***n***	***n***		

2	15	13	9	11	0.00	1.000

4	14	7.5	11	6	6.98	0.008

6	14	4.5	11	2.5	21.5	**< 0.001**

8	8	2	12	6.5	9.55	**0.002**

10	5.5	2	7.5	1.5	11.64	**< 0.001**

†The change in number of females on two blood feeders with contrasting bloodmeal quality (salted and normal blood) used as reinforcing stimuli associated with and without Citronella smell for bi-directional conditioning. The position of each feeder was alternated every 2 min (see methods for details). Significant results are in bold

**Figure 5:**
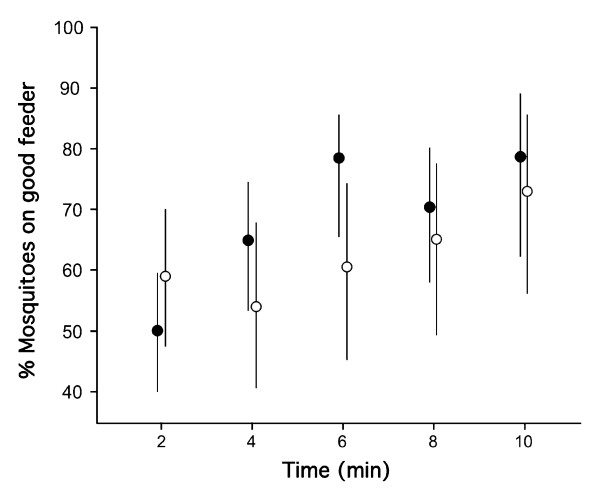
**Effect of different types of unpaired olfactory stimuli on learning rate in *Anopheles gambiae *females**. The mean proportion (± 95%CIs) of females probing and feeding on the preferred feeder associated with the positive reinforcement stimulus of non-salted blood is shown for two sets of experiments. In the first experiment (white circles) the olfactory cue of Blue Shropshire cheese smell was associated with one of the feeder and the other feeder was kept odourless or *vice-versa*; in the second (black circles), Citronella smell was used in a similar way (see methods for details).

Comparing the effect of different innate unpaired cue - i.e. the attractive cheese smell versus Citronella - using logistic regression, revealed significantly better learning when using Citronella rather than cheese (Logistic regression: n = 557, time: ***χ*^2^**= 16.9, *df*= 4, *p*= 0.002; stimulus type: ***χ*^2^**= 12.2, *df*= 1, *p *< 0.001, replicate: ***χ*^2^**= 31.08, *df*= 6, *p *< 0.001) (Figure 5).

### Long-term olfactory memory

Whether trained females remembered the association between the attractive Blue Shropshire cheese smell and salted or non-salted blood 72 h after the initial training/learning session was tested by conducting a second session and then comparing the female initial distribution on the feeders at 2 min in the two sessions (two independent experiments replicated two times in each direction). No statistical difference between the two sessions was found and the main determinant of female distribution on the feeders was the association of cheese smell with the feeder with non-salted blood (Logistic regression: session: *n*= 123, session: ***χ*^2^**= 0.09, *df*= 1, *P*= 0.768, association: ***χ*^2^**= 8.40, *df*= 1, *p*= 0.004, interaction ns).

The same analyses, was used to test for long-term memorization of the association between the disliked Citronella stimulus and salted/non-salted blood. Across the two sessions, females preferred the feeder with non-salted blood but this time that preference was independent of the direction of the association. Females significantly remembered the association after 72 h as evidenced by the significant effect of session on the number of females on each feeder (Logistic regression: *n*= 192, session: ***χ*^2^**= 4.77, *df*= 1, *P*= 0.029, association: ***χ*^2^**= 0.01, *df*= 1, *p*= 0.970, interaction ns).

## Discussion

### Visual memory

This study reports strong evidence of short-term memory in response to conditioning with visual (chequered and white patterns) cues. To our knowledge, this is the first study that experimentally conditioned mosquitoes into associating visual patterns with a reinforcing stimulus, here bloodmeal quality, thereby demonstrating the occurrence of visual memory. Interestingly and predictably, learning rate depended on the strength of the bi-directional positive and negative reinforcing stimuli. In a first series of experiments, the preferred sausage skin membrane and the less liked Parafilm were used as reinforcing stimuli, which resulted in learning but at a slow rate. In several instances it took more than the five intervals (10 min) to train all mosquitoes, leaving more opportunities to have spurious results in the last interval generated by a few late-feeding females. This slower learning rate is not surprising because the negative reinforcing stimulus of the Parafilm still equated to a successful bloodmeal, hence a profitable experience for feeding females. Adding salt to the blood allowed for a truly negative reinforcing stimulus as feeding was prevented altogether due to bloodmeal unpalatability. This resulted in a much stronger differential conditioning and faster learning. Indeed the distribution of females on both feeders suggested that a single attempt at feeding on the salted blood may have led to effective conditioning. The use of salt here had the same effect as the quinine used in previous studies on other insect species (e.g. [7,14,22]). Another factor that significantly affected learning was the 'direction' of the association between unpaired and reinforcing. Mosquitoes prefer darker locations where they are inconspicuous. As a result, females had an initial innate preference for the feeder with chequered pattern that made them feel safer while feeding, and consequently learned to associate that pattern with the positive reinforcing stimulus of a salt-free bloodmeal significantly faster than when the patterns was associated with salted blood.

That female mosquitoes effectively learned to follow the cues associated with their preferred bloodfeeder was tested several times in the course of this study and simply demonstrated by changing the association between the cue under study and the reinforcing stimuli at the last time interval of an experiment. It is noteworthy that nearly all females were successfully fooled into probing/feeding on bad quality blood in such 'method validation experiments'. These results also enabled us to dismiss the possibility that females would actively sample both feeders before settling on the better tasting one within the time elapsed between mosquito counting on each feeder. *An. gambiae *females seldom change location once they start feeding on a feeder probably because changing biting site carries important costs to feeding females in nature.

Bi-directional conditioning led to long-term visual memory with mosquito females remembering the colour pattern associated pattern for as long as 24 and 72 h but not 96 h. This duration is comparable to that found in a bi-directional conditioning experiments conducted on carpenter ants [14]. It is worth pointing out that in honeybee studies that have examined those aspects in detail, long-term association retention usually depended on the number of conditioning trials [27], but also on stimulus strength [28]. In preliminary experiments (results not shown), it was not found to be possible to generate visual memory retrieval when using the moderate differential stimuli of membrane quality (see above), however experiments using salted blood led to successful visual memory retention. One potential biological explanation for the three-day retention duration could be that spatial memory associated with a bloodmeal is linked to the context of host finding and feeding in mosquito females, hence depends on their gonotrophic cycle which takes two to two-and-half days in the wild [29,30]. Spatial memory would help females return to a feeding location [31] or to a host on which they fed successfully in their previous gonotrophic cycle, as suggested by some studies [19]. This would thus minimize the costs associated with host finding and maximize foraging efficiency.

### Olfactory memory

Importantly, similar results using olfactory cues were found, albeit those results strongly depended on the type of odour used. Females learned to associate both Blue Shropshire cheese and Citronella smells. However, there was such a strong innate attraction for the cheese smell that learning was significantly hindered when the cheese smell was associated with salted blood. When Citronella was used, the direction of the association between presence/absence of odour and salted/non-salted blood did not affect learning significantly. This may seem to contradict evidence showing that Citronella is an effective mosquito repellent [26,32]. However formulation and the mode of application are important for effectiveness; and here, because females did not have to be in direct contact with Citronella essence, this may have decreased its repellency.

Importantly, learning was achieved at an overall faster rate with Citronella than with cheese because of the similarity in learning efficiency regardless of the direction of the association between odour and reinforcing stimuli. This had drastic consequences for long-term olfactory memory retention, as significant memory retrieval could not be detected following the partial conditioning achieved with cheese odour but females remembered the associations with Citronella smell for 72 h. No attempt were made to prolong the training/conditioning session with cheese until all females learned to avoid the smell of cheese, but it is unlikely that this would have improved memory retention, such is their innate attraction to some cheese varieties [25].

The fact that females could retain memory of an association between odour and a negative physiological experience such as probing/tasting very salty blood suggest that associative learning could potentially play a role in behavioural avoidance of pesticides. If host-seeking females encounter sub-lethal doses of insecticide whilst seeking hosts, there is certainly no reason why they should not learn to associate their smell and ill effects and subsequently avoid these pesticides when they smell them. Thus the exact role and importance of learning and memory in insecticide excito-repellency remain to be investigated. A role for learning and memory could generally also be expected in devices or vector control strategies that would make use of attractants (visual or olfactory) in combination with a vector killing system that does not provide full lethality, such as sticky traps or exposure to partially lethal pathogens.

### Methodological limitations

The bi-directional dual-feeder conditioning experimental cage set-up developed for these studies enabled to condition groups of female mosquitoes. This was a major improvement over the individual-based approach previously used in studies of associative memory in adult mosquitoes [22], cockroaches [7], or ants [14]. This novel approach enabled to quickly build-up sample sizes, compare experimental groups or replicates; hence explore relatively quickly more complex processes of associative memory.

The downside of such approach is the lack of real control over which females were actually exposed to the contrasted bloodmeal quality/visual or olfactory cues association because they cannot all be forced to feed and participate in the experiments. Thus varying amounts of variation in the analyses were attributable to varying number of females that were not hungry at the time of the conditioning experiments. Other females initiated their feed after a certain delay, hence joined the experiment near its end, thereby creating spurious background noise (e.g. Figure 3 at 10 min). Mosquito rearing was thus critical in ensuring that larvae were reared under standard conditions and that they resulted in adults of homogenous quality and behaviour. This helped ensure that a majority of females fed at the start of the training/testing session, which is critical for most experiments but particularly for long-term memory ones in which females were re-tested in the following days.

## Conclusions

The results show that *Anopheles gambiae *females were capable of associating visual and olfactory stimuli with a positive or negative reinforcing stimulus after only one or two conditioning exposures to that association. This resulted in short-term but also long-term memory with a maximum retention time of 72 h. Learning depended on the strength of the reinforcing stimulus used and, when innate preferences to a visual or olfactory cue were involved, on the direction of the association between the two visual or olfactory stimuli and the positive and negative reinforcing stimuli used for bi-directional conditioning. The possibility that host-seeking females would return to preferred memorized feeding locations and hosts confirms the potential importance of memory for mosquito optimal foraging and suggest that it may also enhance underlying heterogeneities in host exposure to bites. The generally understated importance of learning for mosquito vectorial capacity and the epidemiology of the diseases they transmit is particularly obvious in the light of its potential role in acquired behavioural avoidance to insecticides.

## Competing interests

The authors declare that they have no competing interests.

## Authors' contributions

NC, EP and FT planned the experiments and analysed the data. NC and EP conducted the experiments. FT wrote the manuscript. All authors read and approved the final manuscript.
